# Quantification and deconvolution of asymmetric LC-MS peaks using the bi-Gaussian mixture model and statistical model selection

**DOI:** 10.1186/1471-2105-11-559

**Published:** 2010-11-12

**Authors:** Tianwei Yu, Hesen Peng

**Affiliations:** 1 Department of Biostatistics and Bioinformatics, Rollins School of Public Health, Emory University, Atlanta, GA, USA

## Abstract

**Background:**

Liquid chromatography-mass spectrometry (LC-MS) is one of the major techniques for the quantification of metabolites in complex biological samples. Peak modeling is one of the key components in LC-MS data pre-processing.

**Results:**

To quantify asymmetric peaks with high noise level, we developed an estimation procedure using the bi-Gaussian function. In addition, to accurately quantify partially overlapping peaks, we developed a deconvolution method using the bi-Gaussian mixture model combined with statistical model selection.

**Conclusions:**

Using extensive simulations and real data, we demonstrated the advantage of the bi-Gaussian mixture model over the Gaussian mixture model and the method of kernel smoothing combined with signal summation in peak quantification and deconvolution. The method is implemented in the R package apLCMS: http://www.sph.emory.edu/apLCMS/.

## Background

Liquid chromatography-mass spectrometry (LC-MS) is one of the major techniques in metabolomics [1-4], as well as a key component in MS-based proteomics [5,6]. The pre-processing of LC-MS data involves a complex workflow including noise reduction, peak identification and quantification, retention time correction, peak alignment and weak signal recovery [7,8]. We have previously reported the apLCMS package which carries out the entire workflow with new algorithms specifically designed for LC-MS data with high mass resolution [9]. High-resolution mass spectrometry, such as Fourier transform mass spectrometry (FT-MS), allows the separation of m/z values at or below 10 ppm level [10], resulting in good separation between metabolites. The high resolution facilitates the use of empirical peak shape models to accurately quantify peaks, which is critical in biomarker studies where the relative quantities of metabolites are compared across samples.

Currently, LC-MS peaks are quantified either by summation of ion count, or using symmetric peak shape models, such as the Gaussian function [7-9]. Both methods have serious drawbacks. The method of ion count summation results in biased quantification when the ion trace has missing intensities, which often occurs in high-resolution LC-FTMS data. The Gaussian peak model can result in bias in peak location estimation and peak quantification when the peaks are asymmetric. Hence asymmetric peak models are necessary for the accurate quantification and identification of metabolites. In addition, some metabolites may share m/z and partially overlap in retention time, which necessitates the development of deconvolution procedures.

A large number of empirical peak shape models have been developed for asymmetric peaks in chromatography, most of which were summarized by Di Marco and Bombi [11]. For a few of the models, advanced deconvolution procedures are available [12-17]. Examples include the non-linear deconvolution based on Powell's method [18] for the polynomial-modified Gaussian (PMG) model [16,19], regression-based methods for the parabolic-Lorentzian modified Gaussian (PLMG) model [17], and various deconvolution methods for the exponentially modified Gaussian (EMG) model [12,13].

**Figure 1:**
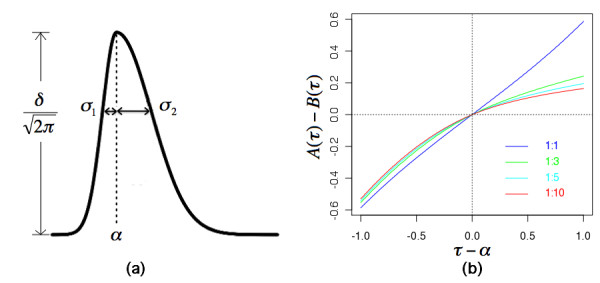
**The characteristics of the bi-Gaussian function**. (a) the four parameters that define the bi-Guassian function; (b) The function *A*(*τ*)-*B*(*τ*) used in our estimation. Different σ_1_/σ_2 _ratios are plotted.

The estimating procedures for asymmetric peak models in chromatographic data generally assume low noise level. In LC-MS data, the noise level is magnitudes higher, and the intensity observations are obtained at much fewer time points. Thus a simple, robust model that can be fitted using a limited number of intensity observations is necessary. The bi-Gaussian peak model (Figure 1a) has been described in the context of chromatography [11,20]. Empirical and theoretical results have shown that the bi-Gaussian model is well suited for asymmetric peaks [20,21]. With four parameters and a simple functional form that's amenable to maximum likelihood estimation, the bi-Gaussian model is suitable for LC-MS data. A parameter estimation method for the bi-Gaussian model has been developed in the openMS environment [22]. The method relies on the observed maximum intensity for the determination of the peak summit location, which could lead to inaccurate estimates when the signal-to-noise ratio is low. Currently no deconvolution method is available for the bi-Gaussian mixture model.

In this paper, we first develop a new algorithm to fit the bi-Gaussian function to noisy ion traces. Secondly, we develop a deconvolution procedure for partially overlapping peaks using the bi-Gaussian mixture model. Thirdly, the low signal-to-noise ratio causes uncertainty in the number of components of the mixture model. We address this issue by a procedure involving statistical model selection. All the algorithms described here have been implemented to improve the apLCMS package for high-resolution LC-MS data analysis [9].

## Methods

### The bi-Gaussian peak model

The model involves four parameters - the location of the peak summit *α*, the standard deviation of the half Gaussian function to the left of the summit *σ*_1_, the standard deviation of the half Gaussian function to the right of the summit *σ*_2_, and the scaling factor *δ *(Figure 1a). The intensity as a function of retention time is modeled by:

$$g\left(t\right)=\{\begin{array}{c} \frac{\delta }{\sqrt{2\pi }}e^{-\frac{{\left(t-\alpha \right)}^{2}}{2\sigma_{1}^{2}}},\;t<\alpha \\ \frac{\delta }{\sqrt{2\pi }}e^{-\frac{{\left(t-\alpha \right)}^{2}}{2\sigma_{2}^{2}}},\;t\geq \alpha \end{array}$$

The areas of the two regions to the left/right of the peak summit are *δσ*_1_/2 and *δσ*_2_/2, respectively.

### The estimation procedure for a single peak

For the estimation of the parameters from the observed data, the most important is to find the peak summit *α*. When the data is noisy, we cannot rely on the observed high point as the estimate. Rather, information from the entire ion trace must be used to estimate the parameter. We define two quantities as a function of retention time *τ*. The first one is the log-ratio of the areas to the left- and right- of *τ*:

$$\begin{array}{l} A\left(\tau \right)\\ =\log\left[\int_{-\infty }^{\tau }g(t)dt\right]-\log\left[\int_{\tau }^{\infty }g(t)dt\right] \end{array}$$

The second quantity is the log-ratio of the cube-root of the non-centered second moments of the left- and right- truncated portions of the function:

$$\begin{array}{l} B\left(\tau \right)\\ =\frac{1}{3}\log\left(\int_{-\infty }^{\tau }g(t){\left(t-\tau \right)}^{2}dt\right)\\ \;\;-\frac{1}{3}\log\left(\int_{\tau }^{\infty }g(t){\left(t-\tau \right)}^{2}dt\right) \end{array}$$

When *τ = α*, the quantity *A*(*α*) is the log ratio between the areas of the two half Guassian functions, which is equal to the log ratio between the two standard deviations; *B*(*α*) is the log ratio between the cubic roots of the variances of the two Gaussian functions multiplied by their scaling factors, which is also equal to the log ratio between the two standard deviations. Thus *τ = α *is a root for *A*(*τ*)-*B*(*τ*) = 0.

$$\begin{array}{l} A\left(\alpha \right)\\ =\log\left(\delta \sigma_{1}/2\right)-\log\left(\delta \sigma_{2}/2\right)\\ =\frac{1}{3}\log\left(\frac{\delta \sigma_{1}^{3}}{2}\right)-\frac{1}{3}\log\left(\frac{\delta \sigma_{2}^{3}}{2}\right)\\ =B\left(\alpha \right) \end{array}$$

Simulations using a reasonable range of *σ*_1_/*σ*_2 _showed that *A*(*τ*)-*B*(*τ*) is a monotone function (Figure 1b), which indicates the solution is unique.

In LC-MS data, the intensity values {*x_1_*,*x*_2_, ..., *x_n_*} are collected at discrete time points {*t_1_*,*t*_2_, ..., *t_n_*}, which means the function *g(t) *is approximated by a step function. We first define the step sizes of the function:

$$\Delta t_{i}=\{\begin{array}{c} t_{2}-t_{1}\text{, }i=1\\ \left(t_{i+1}-t_{i-1}\right)/2\text{, }1<i<n\\ t_{n}-t_{n-1}\text{, }i=n \end{array}$$

We approximate *A*(*τ*) by

$$\begin{array}{l} \hat{A}\left(\tau \right)\\ =\log\left[\sum_{t_{i}<\tau }x_{i}\Delta t_{i}\right]-\log\left[\sum_{t_{i}\geq \tau }x_{i}\Delta t_{i}\right] \end{array}$$

And *B*(*τ*) by

$$\begin{array}{l} \hat{B}\left(\tau \right)\\ =\frac{1}{3}\log\left[\sum_{t_{i}<\tau }x_{i}{\left(t_{i}-\tau \right)}^{2}\Delta t_{i}\right]\\ \;\;-\frac{1}{3}\log\left[\sum_{t_{i}\geq \tau }x_{i}{\left(t_{i}-\tau \right)}^{2}\Delta t_{i}\right] \end{array}$$

Because the data are generated from discrete time points, we first find $\hat{A}\left(\tau \right)-\hat{B}\left(\tau \right)$ for all the middle points between adjacent *t's*. Then we interpolate between the largest point below zero and the smallest point above zero to find $\hat{\alpha }$. After finding $\hat{\alpha }$, estimating *σ*_1 _and/*σ*_2 _becomes straight-forward:

$$\begin{array}{l} {\hat{\sigma }}_{1}=\sqrt{\sum_{t_{i}<\hat{\alpha }}{\left(t_{i}-\hat{\alpha }\right)}^{2}x_{i}\Delta t_{i}/\sum_{t_{i}<\hat{\alpha }}x_{i}\Delta t_{i}}\\ {\hat{\sigma }}_{2}=\sqrt{\sum_{t_{i}\geq \hat{\alpha }}{\left(t_{i}-\hat{\alpha }\right)}^{2}x_{i}\Delta t_{i}/\sum_{t_{i}\geq \hat{\alpha }}x_{i}\Delta t_{i}} \end{array}$$

To estimate the scaling factor *δ*, we first find the fitted values without scaling:

$${\hat{z}}_{i}=\{\begin{array}{c} \frac{1}{\sqrt{2\pi }}e^{-\frac{{\left(t_{i}-\hat{\alpha }\right)}^{2}}{2{\hat{\sigma }}_{1}^{2}}},\;t_{i}<\hat{\alpha }\\ \frac{1}{\sqrt{2\pi }}e^{-\frac{{\left(t_{i}-\hat{\alpha }\right)}^{2}}{2{\hat{\sigma }}_{2}^{2}}},\;t_{i}\geq \hat{\alpha } \end{array}$$

Then the estimate $\hat{\delta }$ is found by a weighted average of the ratio between the observed intensities and the fitted values without scaling. Because ion counts are highly skewed, the calculation is carried out in log scale, giving higher weights to points closer to the summit of the curve,

$$\hat{\delta }=e^{\sum_{i}{\hat{z}}_{i}^{2}\times \log\left(x_{i}/{\hat{z}}_{i}\right)/\sum_{i}{\hat{z}}_{i}^{2}}$$

### Fitting the bi-Gaussian mixture model

In LC-MS data from complex samples, e.g. serum or urine, sometimes peaks sharing m/z value may also partially overlap in the retention time dimension. Here we propose an EM-like iterative algorithm to fit partially overlapping asymmetric peaks. The expectation-maximization (EM) algorithm finds maximum likelihood estimates of parameters in the presence of latent variables. It iterates between finding the expectation of the log-likelihood with regard to the latent variables given the current estimate of the parameters, and finding the parameters that maximize the likelihood [23]. In our application, the parameter estimation is not obtained using the maximum likelihood procedure, and an extra step of eliminating components that explain too small a proportion of the data is added to deal with the noise.

(1) Fit a kernel smoother to the data {(*t_i_*,*x*_i_)}. Split the data points into groups at the valleys of the smoother. For every group *j *of the data points, use the smoother peak as the initial estimate of peak summit ${\hat{\alpha }}_{j}$, and estimate ${\hat{\sigma }}_{j,1}$, ${\hat{\sigma }}_{j,2}$, and ${\hat{\delta }}_{j}$ using the procedure in the previous sub-section. More discussion about smoother parameter selection is presented in the next sub-section.

(2) Iterate until convergence:

(2.1) Find the fitted values at every *t_i _*for component *j*,

$${\hat{z}}_{ij}=\{\begin{array}{c} \frac{{\hat{\delta }}_{j}}{\sqrt{2\pi }}e^{-\frac{{\left(t_{i}-{\hat{\alpha }}_{j}\right)}^{2}}{2{\hat{\sigma }}_{j,1}^{2}}},\;t_{i}<{\hat{\alpha }}_{j}\\ \frac{{\hat{\delta }}_{j}}{\sqrt{2\pi }}e^{-\frac{{\left(t_{i}-{\hat{\alpha }}_{j}\right)}^{2}}{2{\hat{\sigma }}_{j,2}^{2}}},\;t_{i}\geq {\hat{\alpha }}_{j} \end{array},\forall i,j$$

(2.2) For every component *j*, find the proportion of data explained by the component:

$$Q_{j}=\frac{\sum_{i}{\hat{z}}_{ij}}{\sum_{k}\sum_{i}{\hat{z}}_{ik}}$$

Remove component *j *if *Q_j _*is smaller than a threshold.

(2.3) For every time point, we find the expected proportion of the observed intensities that belong to each component *j*, denoted *q_ij_*.

$$q_{ij}=\frac{{\hat{z}}_{ij}}{\sum_{k}{\hat{z}}_{ik}},\forall i,j$$

Then for every component *j*, re-estimate $\left\{{\hat{\alpha }}_{j},{\hat{\sigma }}_{j,1},{\hat{\sigma }}_{j,2},{\hat{\delta }}_{j}\right\}$ from the data {(*t_i_*,*x_i_q_ij_*)}, using the procedure described in the previous sub-section.

### Choosing the number of components of the mixture by statistical model selection

In the previous sub-section, the kernel smoother is employed to obtain an initial estimate of the number of components and the parameters. When the data is noisy, changing the window size of the kernel smoother could result in different numbers of components of the mixture. To find the best model to explain the data, we utilize statistical model selection based on the Bayesian information criterion (BIC) [24]. BIC is one of the most popular criteria for the selection among a set of parametric models with different number of parameters. It penalizes the number of free parameters. The model with lower BIC value is preferred.

First, a reasonable range of the window-size parameter is determined based on biological/chemical considerations about potential peak width. It can be quite lenient to cover a wide range of potential values. Several window size values spanning the range are selected. Starting from each of the window-size value, we compute the kernel smoother, and run the EM-like algorithm described in the previous sub-section. The corresponding BIC value is computed by:

$$\begin{array}{l} N\times \log\left[\left(\sum_{i}{\left(x_{i}-\sum_{j}{\hat{z}}_{ij}\right)}^{2}\right)/N\right]\\ +4\times J\times \log\left(N\right) \end{array}$$

where *N *is the total number of time points with observed intensities, and *J *is the number of bi-Gaussian components in the model. The model with the lowest BIC value is selected. In the setting of LC-MS data, this is a heuristic criterion, because the data we observe are not random samples, and the Gaussian error assumption of BIC may not be satisfied. We justify the usage of the criterion by extensive simulations.

### Simulations

To assess the performance of the proposed method, extensive simulations were conducted. The bi-Gaussian mixture model with BIC model selection was compared with two other methods - the Gaussian mixture model [9] with BIC model selection, and the peak quantification based on kernel smoother and signal summation.

The data were generated from a 3-component bi-Gaussian mixture model, with different levels of peak asymmetry, noise and peak overlap. Given the parameters (Additional file 1: Table S1), the data from each component are generated from the bi-Gaussian functions:

$$g_{j}\left(t\right)=\{\begin{array}{c} \frac{\delta_{j}}{\sqrt{2\pi }}e^{-\frac{{\left(t-\alpha_{j}\right)}^{2}}{2\sigma_{j,1}^{2}}},\;t<\alpha_{j}\\ \frac{\delta_{j}}{\sqrt{2\pi }}e^{-\frac{{\left(t-\alpha_{j}\right)}^{2}}{2\sigma_{j,2}^{2}}},\;t\geq \alpha_{j} \end{array}$$

After summing the intensities from the components, multiplicative noise was added to the data. In addition, a portion of the values were turned into zero to mimic the behavior of real high-resolution LC-MS data:

xi=∑jgj(ti)×eεi×ui,εi~N(0,ξ),ui~binom(θ)

The parameter *ξ *is the standard deviation of the noise added at the log-scale. Three levels of *ξ *were used in the simulations (0.2, 0.4, 0.6). At the high noise level of *ξ *= 0.6, 50% of the intensity values were changed by 1.5 fold or more, and 25% were changed by two fold or more. The parameter *θ *controls the percentage of values turned into zero using random samples from the binomial distribution. Three levels of *θ *were used (0, 0.25, 0.5). The value of *θ *directly corresponds to the proportion of intensities turned into zero. In addition, various levels of peak asymmetry and overlap were considered (Additional file 1: Table S1). In total 864 parameter combinations were tested. At each parameter setting, the simulation was performed 100 times. For detailed information, please refer to Additional file 1.

## Results

### Simulation results

**Figure 2:**
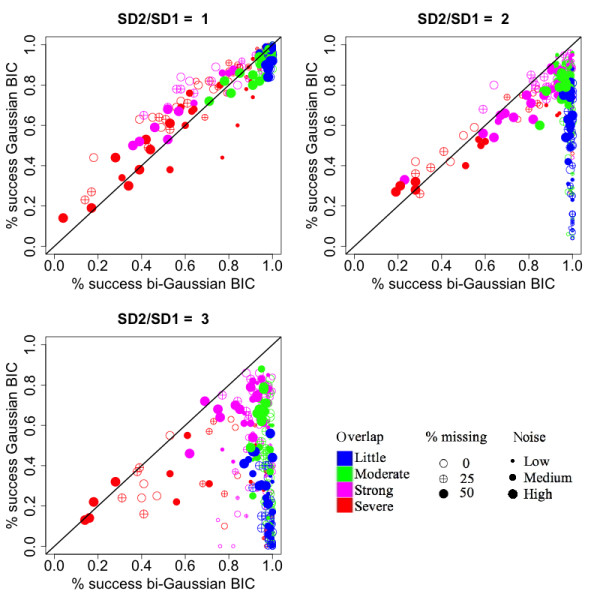
**Comparison of the rate of successfully selecting the correct number of components between the bi-Gaussian mixture model and the Gaussian mixture model**. Each sub-plot corresponds to a different degree of asymmetry, as shown in the titles of the sub-plots (ratios between the right- and left- standard deviations). Each dot represents a simulated situation. The values were obtained by averaging the results from 100 simulations. The color represents the level of overlaps between the simulated peaks. The size of the dot represents the amount of noise added to the data. The fill of the dot represents the percentage of values missing in the ion trace.

First, we compared the rate of successfully selecting the correct number of components between the bi-Gaussian mixture model and the Gaussian mixture model (Figure 2). The method of kernel smoother combined with signal summation wasn't compared because no BIC model selection could be performed using this method, which is a shortcoming in itself. In summarizing the results, the level of peak overlap is defined by the ratio *r *between the lowest point of the valley between two peaks and the lower of the peak summits, before noise is introduced. Because two valleys exist between the three simulated peaks, the larger *r *value is taken for each simulation setting. For the purpose of plotting, we roughly divide the amount of overlap into four categories: little overlap (r < 0.2), moderate overlap (0.2 ≤ r < 0.5), strong overlap (0.5 ≤ r < 0.75), and severe overlap (r ≥ 0.75). The level of overlapping is color-coded. The point size corresponds to the three levels of noise added to the data (*ξ *= 0.2,0.4, 0.6). The fill of the point represents the proportion of missing values (0%, 25% and 50%).

When the peaks were symmetric (Figure 2, upper-left panel), the Gaussian mixture model showed a slight advantage when the overlapping was strong (red and magenta points). When the peaks were asymmetric (Figure 2, upper-right and lower-left panels), the bi-Gaussian mixture model showed a clear advantage. When the peak overlapping was not strong (blue and green points), the success rate of the bi-Gaussian mixture model was mostly higher than 90%, even when the noise level was high. When there was strong peak overlapping and the noise level was high (larger sized red and magenta points), the rate of successfully selecting the correct number of components was reduced for both the bi-Gaussian mixture model and the Gaussian mixture model.

**Figure 3:**
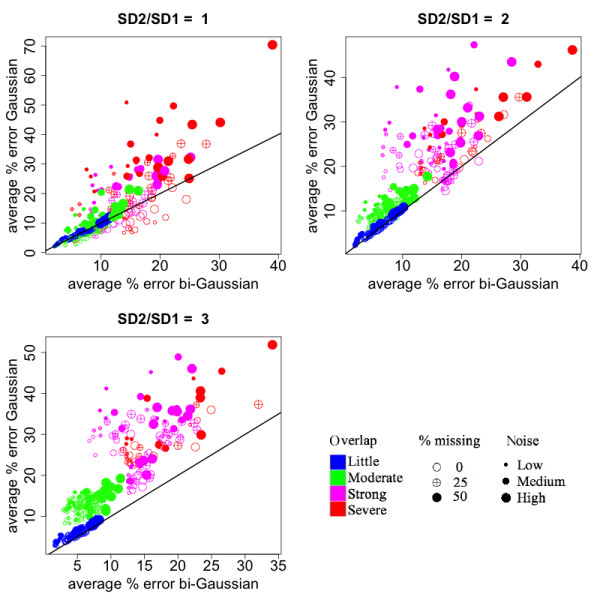
**Comparison of the accuracy in peak size quantification between the bi-Gaussian mixture model and the Gaussian mixture model**. Each sub-plot corresponds to a different degree of asymmetry, as shown in the titles of the sub-plots (ratios between the right- and left- standard deviations). Each dot represents a simulated situation. The values were obtained by averaging the results from 100 simulations. The color represents the level of overlaps between the simulated peaks. The size of the dot represents the amount of noise added to the data. The fill of the dot represents the percentage of values missing in the ion trace.

**Figure 4:**
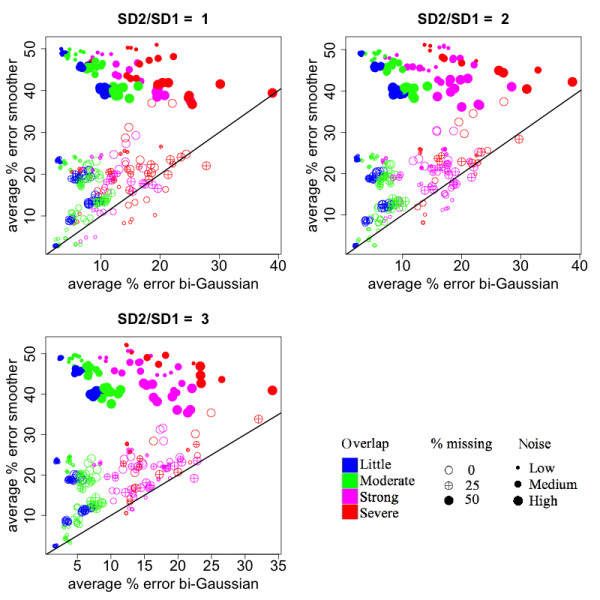
**Comparison of the accuracy in peak size quantification between the bi-Gaussian mixture model and the method of kernel smoother combined with signal summation**. Each sub-plot corresponds to a different degree of asymmetry, as shown in the titles of the sub-plots (ratios between the right- and left- standard deviations). Each dot represents a simulated situation. The values were obtained by averaging the results from 100 simulations. The color represents the level of overlaps between the simulated peaks. The size of the dot represents the amount of noise added to the data. The fill of the dot represents the percentage of values missing in the ion trace.

Secondly, we compared the percentage error in peak area quantification between the three methods, when all three methods were able to identify the correct number of components (not necessarily the best BIC value). Compared to the Gaussian mixture model, the bi-Gaussian mixture model yielded much smaller errors when the peaks were asymmetric (Figure 3, upper-right and lower-left panels). Compared to the method of kernel smoother combined with signal summation, the bi-Gaussian mixture model showed a clear advantage when some of the intensity values were missing (filled points) (Figure 4). When the peak overlapping was not strong (blue and green points), the error of the bi-Gaussian mixture model was mostly under 15%. Further comparisons on peak location and peak spread estimation are presented in Additional file 1. The bi-Gaussian mixture model also clearly out-performed the other two methods in those aspects (Additional file 1: Fig. S2~S4).

### Analysis of high-resolution LC-MS data

**Figure 5:**
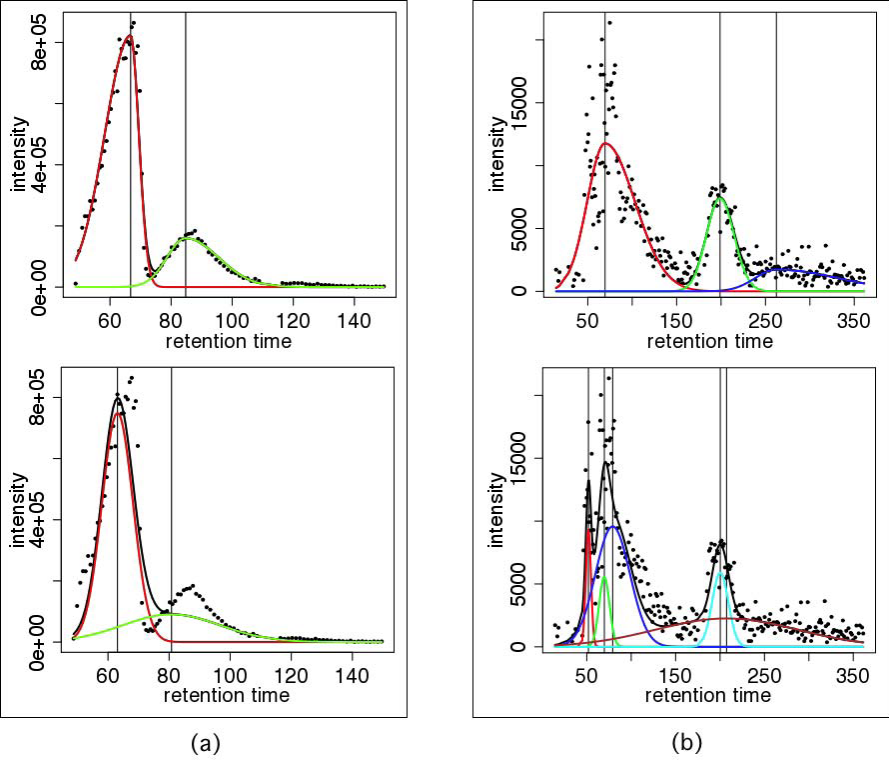
**Comparison of the fit of the bi-Gaussian mixture model and the Gaussian mixture model to real asymmetric peaks**. (a) The ion trace at m/z = 446.8913. (b) The ion trace at m/z = 301.1409. Colored curves: fitted components; black curve: summation of the signal from all the components. Upper-panel: the bi-Gaussian mixture fit; lower-panel: the Gaussian mixture fit.

We implemented the new algorithms in the apLCMS package for LC-MS metabolomics data analysis [9]. When analyzing the example dataset at the apLCMS website, which contains 8 high-resolution LC-MS profiles, we observed many examples where the peaks were clearly asymmetric. We show two examples in Figure 5, where both peak asymmetry and peak overlapping exist. In both examples, the inability of the Gaussian curve to fit asymmetric peaks left residuals to be fitted by the smaller peaks, which caused the smaller fitted peaks to deviate from the local peak shape (Figure 5, lower panels). Clearly the bi-Gaussian mixture model fitted the data much better (Figure 5, upper panels).

At the global level, in 21.0% of the ion traces, the bi-Gaussian mixture model and the Gaussian mixture model selected different number of components. Among these cases, the bi-Gaussian mixture model fitted the data with smaller number of components 93.7% of the time. In addition, it achieved better BIC scores in 66.2% of the cases. Overall, in 59.4% of all the ion traces, the bi-Gaussian (mixture) model achieved better BIC values compared to the Gaussian (mixture) model. Considering the bi-Gaussian model is penalized more heavily by BIC with the extra parameter, which puts it in disadvantage when the peak is close to symmetric, these results indicate that the bi-Gaussian peak model is indeed better suited for the data.

## Discussions

Compared to the Gaussian peak shape model, which has been used in some model-based data processing pipelines [8,9], the bi-Gaussian model provides extra flexibility to fit asymmetric peaks, while suffering little disadvantage when the true peak shape is symmetric. Compared to the method of kernel smoother combined with signal summation, fitting a bi-Gaussian mixture model disentangles partially overlapping peaks, and copes with the issue of missing intensities in high-resolution LC-FTMS data much better. The bi-Gaussian model is among many asymmetric peak models in chromatographic peak modeling. A large number of other models could potentially be used for the processing of LC-MS data [11]. Advanced deconvolution methods already exist for a few of the models [12-17,19]. However, modifications to the existing estimation procedures may be necessary to suit the characteristics of LC-MS data, i.e. sparser data points and much higher noise.

In this study, the parameter estimation for a single peak is done by numerically solving an equation that involves the zero and second moments of the truncated distribution functions. An alternative route is to use the maximum likelihood method. We developed a likelihood-based algorithm (Additional file 1: Section S4) and compared its performance with the moment-based method in simulations. The likelihood-based algorithm was slower in computation due to its iterative nature, and it didn't achieve better estimation accuracy over the moment-based method. Under the settings of our simulations, five window size values were used for the initiation of the model selection process. With both methods programmed in R, using a single core of a 2.26 GHz Xeon CPU, the median CPU time for solving the three-component mixture was 0.15 second for the moment-based method, and 0.33 second for the likelihood-based method.

## Conclusion

In this manuscript, we presented a method to fit the bi-Gaussian curve to noisy LC-MS ion traces, as well as an EM-like algorithm paired with BIC model selection for the deconvolution of partially overlapping peaks. Currently, the methods were implemented in the apLCMS package for the pre-processing of high-resolution LC-MS data. The same modeling procedure can be adapted easily into other pipelines for the quantification of both metabolites and peptides.

## Authors' contributions

TY designed the study, developed the methods, conducted data analysis, and drafted the manuscript. HP developed the likelihood-based estimation procedure, and drafted the corresponding method description (Additional file 1: Section S4.1). Both authors have read and approved the final manuscript.

## Supplementary Material

Additional file 1 **Supporting Material**. The file contains details of the simulation study, additional results of the simulation study, extra figure illustrating the method workflow, and description of the likelihood-based estimation procedure of the bi-Gaussian model. Click here for file

## References

[B1] Issaq HJ Van QN Waybright TJ Muschik GM Veenstra TD Analytical and statistical approaches to metabolomics research J Sep Sci 2009 32 13 2183 2199 10.1002/jssc.200900152 19569098

[B2] Dettmer K Aronov PA Hammock BD Mass spectrometry-based metabolomics Mass Spectrom Rev 2007 26 1 51 78 10.1002/mas.20108 16921475 PMC1904337

[B3] Dunn WB Current trends and future requirements for the mass spectrometric investigation of microbial, mammalian and plant metabolomes Phys Biol 2008 5 1 11001 10.1088/1478-3975/5/1/011001 18367780

[B4] Griffin JL Kauppinen RA A metabolomics perspective of human brain tumours Febs J 2007 274 5 1132 1139 10.1111/j.1742-4658.2007.05676.x 17298437

[B5] Chen G Pramanik BN Application of LC/MS to proteomics studies: current status and future prospects Drug Discov Today 2009 14 9-10 465 471 10.1016/j.drudis.2009.02.007 19429505

[B6] Ahmed FE Utility of mass spectrometry for proteome analysis: part II. Ion-activation methods, statistics, bioinformatics and annotation Expert Rev Proteomics 2009 6 2 171 197 10.1586/epr.09.4 19385944

[B7] Katajamaa M Oresic M Data processing for mass spectrometry-based metabolomics J Chromatogr A 2007 1158 1-2 318 328 10.1016/j.chroma.2007.04.021 17466315

[B8] Smith CA Want EJ O'Maille G Abagyan R Siuzdak G XCMS: processing mass spectrometry data for metabolite profiling using nonlinear peak alignment, matching, and identification Anal Chem 2006 78 3 779 787 10.1021/ac051437y 16448051

[B9] Yu T Park Y Johnson JM Jones DP apLCMS--adaptive processing of high-resolution LC/MS data Bioinformatics 2009 25 15 1930 1936 10.1093/bioinformatics/btp291 19414529 PMC2712336

[B10] Ahmed FE Utility of mass spectrometry for proteome analysis: part I. Conceptual and experimental approaches Expert Rev Proteomics 2008 5 6 841 864 10.1586/14789450.5.6.841 19086863

[B11] Di Marco VB Bombi GG Mathematical functions for the representation of chromatographic peaks J Chromatogr A 2001 931 1-2 1 30 10.1016/S0021-9673(01)01136-0 11695512

[B12] Felinger A Deconvolution of Overlapping Skewed Peaks Analytical Chemistry 1994 66 19 3066 3072 10.1021/ac00091a013

[B13] Johansson M Berglund M Baxter DC Improving Accuracy in the Quantitation of Overlapping, Asymmetric, Chromatographic Peaks by Deconvolution - Theory and Application to Coupled Gas-Chromatography Atomic-Absorption Spectrometry Spectrochim Acta B 1993 48 11 1393 1409 10.1016/0584-8547(93)80127-G

[B14] Papai Z Pap TL Determination of chromatographic peak parameters by non-linear curve fitting using statistical moments Analyst 2002 127 4 494 498 10.1039/b111304f 12022647

[B15] Youn DY Yun SJ Jung KH Improved Algorithm for Resolution of Overlapped Asymmetric Chromatographic Peaks J Chromatogr 1992 591 1-2 19 29 10.1016/0021-9673(92)80219-K

[B16] TorresLapasio JR GarciaAlvarezCoque MC BaezaBaeza JJ Global treatment of chromatographic data with MICHROM Anal Chim Acta 1997 348 1-3 187 196 10.1016/S0003-2670(97)00066-4

[B17] Caballero RD Garcia-Alvarez-Coque MC Baeza-Baeza JJ Parabolic-Lorentzian modified Gaussian model for describing and deconvolving chromatographic peaks Journal of Chromatography A 2002 954 1-2 59 76 10.1016/S0021-9673(02)00194-2 12058919

[B18] Powell MJD A Method for Minimizing a Sum of Squares of Non-Linear Functions without Calculating Derivatives Comput J 1965 7 4 303 307

[B19] TorresLapasio JR BaezaBaeza JJ GarciaAlvarezCoque MC A model for the description, simulation, and deconvolution of skewed chromatographic peaks Analytical Chemistry 1997 69 18 3822 3831 10.1021/ac970223g

[B20] Buys TS De Clerk K Bi-Gaussian fitting of skewed peaks Analytical Chemistry 1972 44 7 1273 1275 10.1021/ac60315a005

[B21] Felinger A Data Analysis and Signal Processing in Chromatography 1998 1 Amsterdam: Elsevier Science

[B22] Sturm M Bertsch A Gropl C Hildebrandt A Hussong R Lange E Pfeifer N Schulz-Trieglaff O Zerck A Reinert K OpenMS - an open-source software framework for mass spectrometry BMC Bioinformatics 2008 9 163 10.1186/1471-2105-9-163 18366760 PMC2311306

[B23] Dempster AP Laird NM Rubin DB Maximum Likelihood from Incomplete Data Via Em Algorithm J Roy Stat Soc B Met 1977 39 1 1 38

[B24] Schwarz G Estimating Dimension of a Model Ann Stat 1978 6 2 461 464 10.1214/aos/1176344136

