# Supplementary material for: Quantification and deconvolution of asymmetric LC-MS peaks using the bi-Gaussian mixture model and statistical model selection
Source: BMC Bioinformatics. 2010 Nov 12;11:559. doi: 10.1186/1471-2105-11-559 (PMC2993707; doi:10.1186/1471-2105-11-559)
Supplement: Additional file 1 — Supporting Material. The file contains details of the simulation study, additional results of the simulation study, extra figure illustrating the method workflow, and description of the likelihood-based estimation procedure of the bi-Gaussian model. [file 1471-2105-11-559-S1.DOC]

**Supporting materials.**

**S1. Additional figures.**


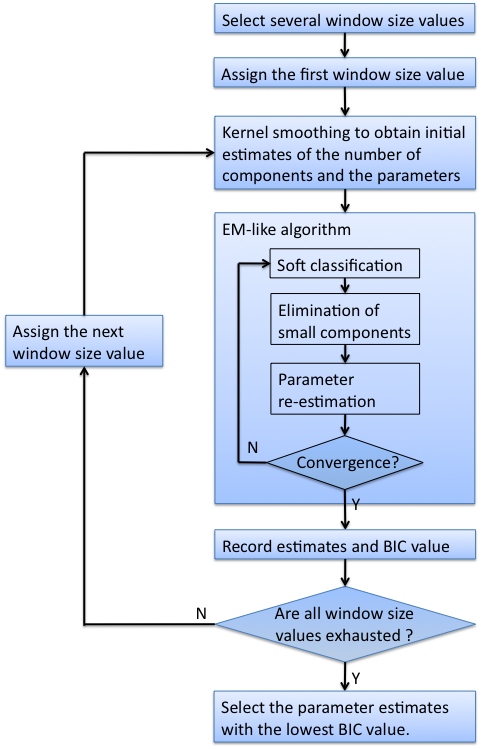


Figure S1. The overall workflow of the BIC-based model selection scheme with the EM-like estimation procedure.

**S2. Simulation setup.**

All data were generated from a 3-component bi-Gaussian mixture model. In 1/3 of the cases, the two standard deviation values of every peak were set equal, causing the bi-Gaussian mixture models to degenerate to Gaussian mixture models. Given the parameters (Table S1), the data from individual components were generated by the bi-Gaussian functions:

$$g_{j}\left(t\right)=\left\{\begin{matrix} \frac{\delta_{j}}{\sqrt{2\pi}}e^{-\frac{\left(t-\alpha_{j}\right)^{2}}{2\sigma_{j,1}^{2}}},\text{ }t<\alpha_{j} \\ \frac{\delta_{j}}{\sqrt{2\pi}}e^{-\frac{\left(t-\alpha_{j}\right)^{2}}{2\sigma_{j,2}^{2}}},\text{ }t\geq\alpha_{j} \end{matrix}\right.$$

After summation, multiplicative noise was added to the data. In addition, a portion of the values were turned into zero to mimic the behavior of real LC/MS data:

We simulated time points from 1 to 800 seconds, with one-second equal intervals. The parameters we used to simulate the intensities are listed in Table S1. With all possible combinations of the parameters, a total of 864 simulation settings were used, each of which was run 100 times.

**Table S1**. The parameter settings used in the simulation study.

| Parameter | Settings |
| --- | --- |
| *α_1_* | *60* |
| *σ_1,1_* | *12* |
| *σ_1,2_* | *σ_1,1_×γ* |
| *δ_1_* | *2000* |
|  |  |
| *α_2_* | *α_1_+(σ_1,2_+σ_2,1_)×b, b=1.5, 2, 3, 4* |
| *σ_2,1_* | *12, 24* |
| *σ_2,2_* | *σ_2,1_×γ* |
| *δ_2_* | *4000* |
|  |  |
| *α_3_* | *α_2_+(σ_2,2_+σ_3,1_)×d, d=1.5, 2, 3, 4* |
| *σ_3,1_* | *12* |
| *σ_3,2_* | *σ_3,1_×γ* |
| *δ_3_* | *3000* |
|  |  |
| *γ* | *1, 2, 3* |
| $\xi$ | *0.2, 0.4, 0.6* |
| $\theta$ | *1, 0.75, 0.5* |

**S3. Comparing the bi-Gaussian mixture model to the Gaussian mixture model and the method of kernel smoother combined with signal summation.**

We compared the bi-Gaussian mixture model with two other methods – the Gaussian mixture model, and the method of kernel smoother combined with intensity summation. For the last method, we first fitted a kernel smoother to the data $\left\{\left(t_{i},x_{i}\right)\right\}$. Then we split the data at the valleys of the smoother. For every group *j*, we used the smoother peak as the estimate of peak summit $\hat{\alpha}_{j}$, and summed the *x’s* of the group as the area estimate. For all three methods, the smoother bandwidth values of 10, 15, 22, 30, 60 were tested.

The original Gaussian mixture model fitting was described in (Yu, et al., 2009), without any model selection procedure. In the current study, BIC model selection was used for both the bi-Gaussian mixture model and the Gaussian mixture model. For the Gaussian mixture model, the BIC was obtained by

,

where *N* is the number of time points with observed intensities, and *J* is the number of components in the model.

Four criteria were used to compare the methods:

1. The frequency that the BIC criterion selects the correct number of components. This comparison was made between the bi-Gaussian and Gaussian mixture models only, because model selection cannot be used in simple signal summation.

When all three models could fit 3 components, using the 3-component fit (not considering the BIC criterion), we compared

1. The percentage error in peak area estimation;
2. The percentage error in peak standard deviation estimation;
3. The absolute error in peak location estimation.

The first two criteria are more critical in real applications.

In summarizing the results, the level of peak overlap is defined by the ratio *r* between the lowest point of the valley between two peaks and the lower of the peak summits, before noise is introduced. Because two valleys exist between the three peaks, the larger value *r* is taken for each simulation setting.

The comparison using the first two criteria are reported in the main text (Fig. 2 ~ 4), which showed clear advantage of the bi-Gaussian mixture model in terms of the success rate of finding the correct number of components and peak area quantification.

Figure S2 shows the comparison in peak location estimation between the bi-Gaussian mixture model and the Gaussian mixture model. The average absolute difference between the identified peak summit and the true peak summit location is plotted. When the peaks were symmetric (upper-left panel), the Gaussian mixture model showed a slight advantage over the bi-Gaussian mixture model. Notice the errors were quite small. When the peaks were asymmetric, the peak location estimation of the Gaussian model was biased towards the longer tail, which explains the big deviations observed in the upper-right panel and the lower-left panel.

| 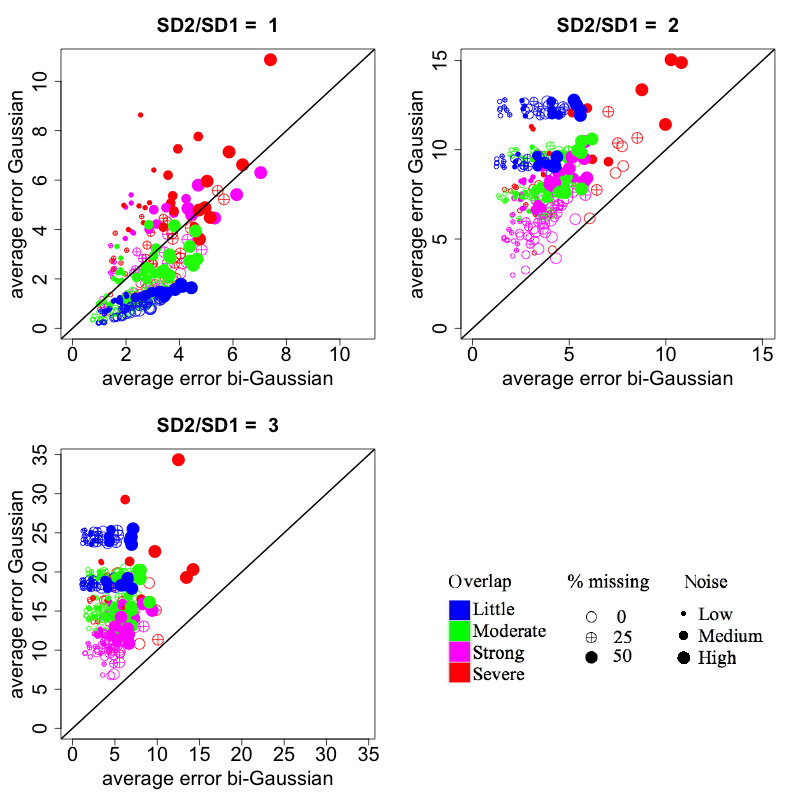 |
| --- |
| Figure S2. Comparison of the accuracy in peak summit estimation between the bi-Gaussian mixture model and the Gaussian mixture model. Each sub-plot corresponds to a different degree of asymmetry, as shown in the titles of the sub-plots (ratios between the right- and left- standard deviations). Each dot represents a simulated situation. The values were obtained by averaging the results from 100 simulations. The color represents the level of overlap between the simulated peaks. The size of the dot represents the amount of noise added to the data. The fill of the dot represents the percentage of values missing in the ion trace. |

Figure S3 shows the comparison in peak location estimation between the bi-Gaussian mixture model and the method of kernel smoother combined with intensity summation. The average absolute difference between the identified peak summit and the true peak summit location are plotted. When the peaks were symmetric (upper-left panel), Both methods performed similarly. Notice the errors were quite small. When the peaks were asymmetric, the method of kernel smoother combined with intensity summation showed larger deviation from the truth, although the difference between the two methods was still relatively small.

| 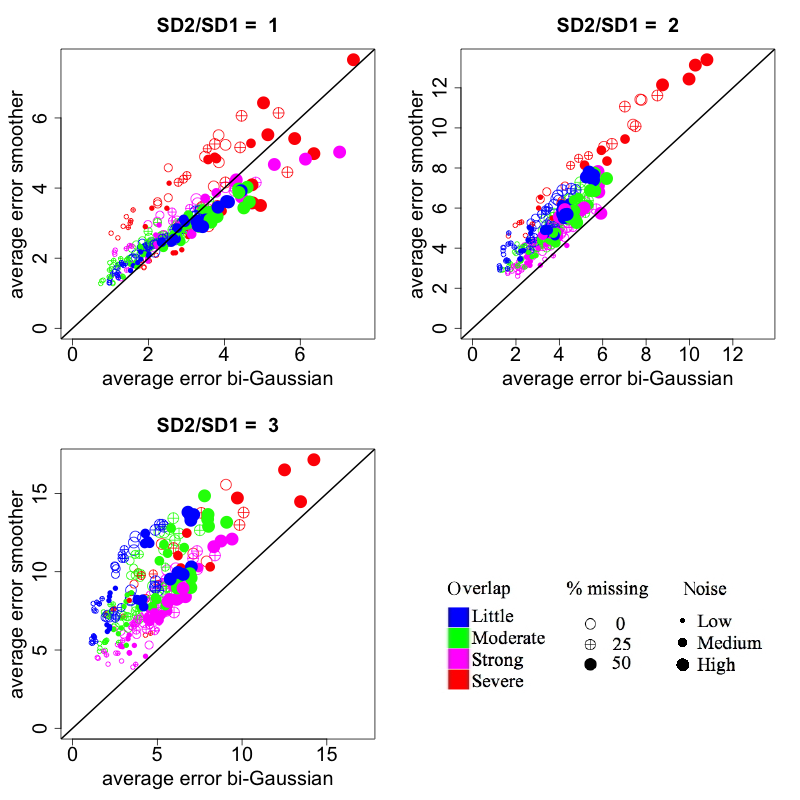 |
| --- |
| Figure S3. Comparison of the accuracy in peak summit estimation between the bi-Gaussian mixture model and the method of kernel smoother combined with intensity summation. Each sub-plot corresponds to a different degree of asymmetry, as shown in the titles of the sub-plots (ratios between the right- and left- standard deviations). Each dot represents a simulated situation. The values were obtained by averaging the results from 100 simulations. The color represents the level of overlap between the simulated peaks. The size of the dot represents the amount of noise added to the data. The fill of the dot represents the percentage of values missing in the ion trace. |

Figure S4 shows the comparison of the estimation accuracy of the standard deviations between the bi-Gaussian mixture model and the Gaussian mixture model. As expected, the Gaussian mixture model showed a slight advantage when the true peaks were symmetric except when the percentage missing was high (upper-left panel), while the bi-Gaussian model clearly out-performed the Gaussian model when the peaks were asymmetric.

| 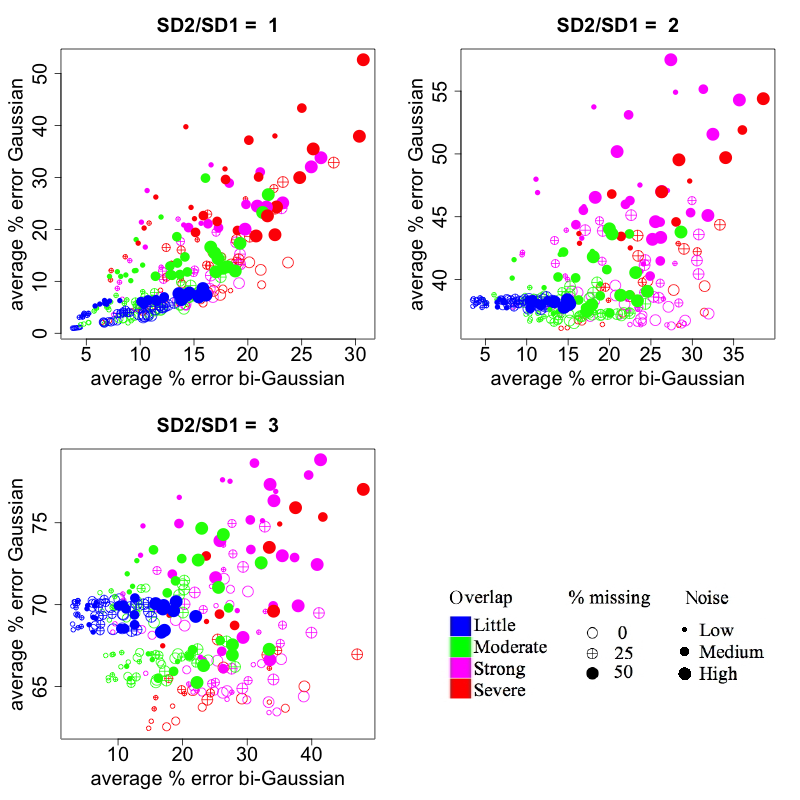 |
| --- |
| Figure S4. Comparison of the accuracy of peak standard deviation estimation between the bi-Gaussian mixture model and the Gaussian mixture model. Each sub-plot corresponds to a different degree of asymmetry, as shown in the titles of the sub-plots (ratios between the right- and left- standard deviations). Each dot represents a simulated situation. The values were obtained by averaging the results from 100 simulations. The color represents the level of overlap between the simulated peaks. The size of the dot represents the amount of noise added to the data. The fill of the dot represents the percentage of values missing in the ion trace. |

**S4. Comparison between moment-based estimation and likelihood-based estimation of the parameters of the bi-Gaussian function.**

Here we compare two estimation methods for the single-peak parameters of the bi-Gaussian function. The first is the moment-based method presented in the main text. The second is the likelihood-based method.

*S4.1 The likelihood-based method.*

Assuming we have observation at time 
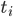
 with weight 
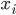
 for 
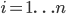
 . And denote the change point location as 
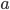
, the standard deviation for each part as 
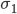
 and 
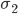
. Then for each observation the probability density function is: 


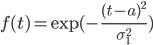
 for 
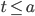
, and 
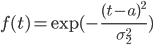
 for 
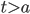
, up to a constant.

With 
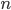
 observations 
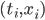
, we have the likelihood function as


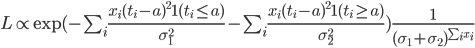


The log-likelihood is
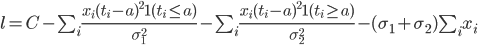


where 
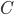
 is a constant independent of 
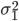
, 
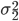
 and 
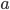
.

The estimate for 
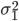
, 
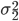
 and 
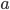
 can be solved iteratively using profile likelihood.

Denote 
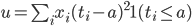
 , 
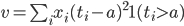
, and 
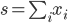
.

1) Given 
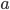
, the log-likelihood becomes


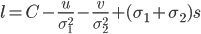


We can get the estimate as 
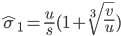


 and 
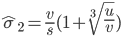
. 

2) Given 
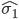
 and 
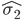
, the log-likelihood becomes
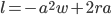
, where 
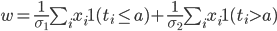
 and
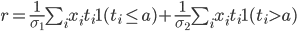
 .

The profile MLE is 
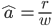
. During the iteration we put the estimate for 
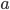
 from the last step into 
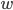
 and 
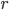
, and obtain new estimate for 
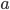
. 
With some reasonable starting value for 
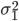
, 
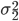
 and 
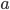
, we can iterate step 1 and 2 until the estimates converge. Since log-likelihood given 
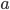
 is concave, we and get consistent estimate for 
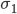
 and 
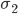
 if we have correct starting point of 
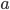
.

*S4.2 Comparison results.*

For the deconvolution of bi-Gaussian mixtures, both methods were integrated into the same EM-like algorithm. Figure S5 shows the comparison of the rate to select the correct number of components. Overall, in most of the cases the moment-based method outperformed the likelihood-based method. When the overlap between peaks was low, both methods performed similarly. When the overlapping was severe (red and magenta points), the moment-based method exhibited some advantage over the likelihood-based method.

| 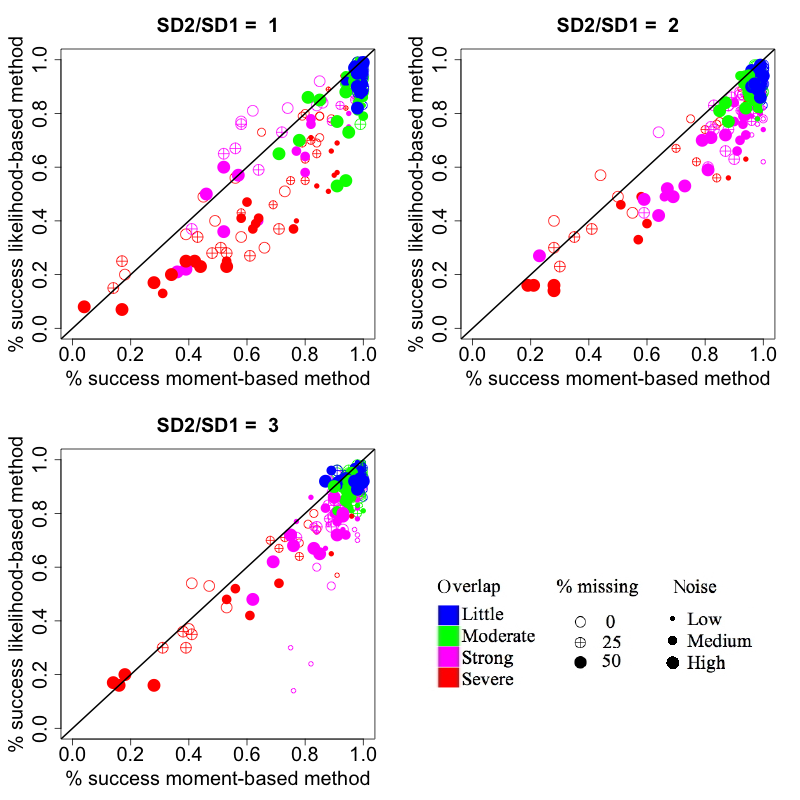 |
| --- |
| Figure S5. Comparison of the rate of successfully selecting the correct number of components between the moment-based estimation procedure and the likelihood-based method. Each sub-plot corresponds to a different degree of asymmetry, as shown in the titles of the sub-plots (ratios between the right- and left- standard deviations). Each dot represents a simulated situation. The values were obtained by averaging the results from 100 simulations. The color represents the level of overlap between the simulated peaks. The size of the dot represents the amount of noise added to the data. The fill of the dot represents the percentage of values missing in the ion trace. |

Figure S6 shows the comparison in peak size quantification. Again the moment-based method maintained a slight edge over the likelihood-based method. The difference appears to be larger when the percentage missing was high (solid dots).

| 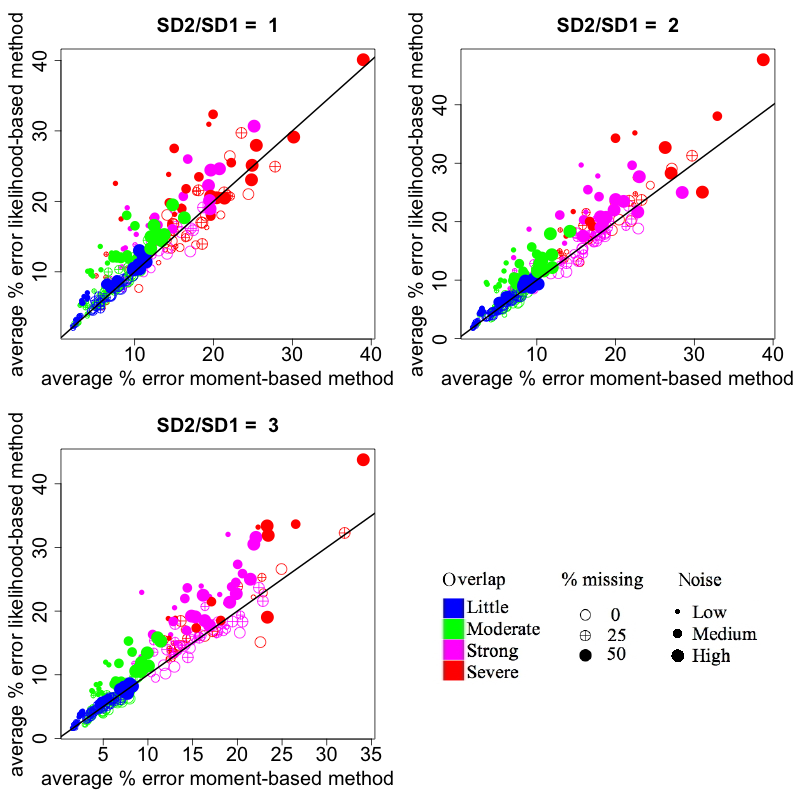 |
| --- |
| Figure S6. Comparison of the accuracy in peak size quantification between the moment-based estimation procedure and the likelihood-based method. Each sub-plot corresponds to a different degree of asymmetry, as shown in the titles of the sub-plots (ratios between the right- and left- standard deviations). Each dot represents a simulated situation. The values were obtained by averaging the results from 100 simulations. The color represents the level of overlap between the simulated peaks. The size of the dot represents the amount of noise added to the data. The fill of the dot represents the percentage of values missing in the ion trace. |

Figure S7 shows the comparison in peak location estimation. Again the moment-based method maintained an edge over the likelihood-based method, especially when the percentage missing was higher (solid dots).

| 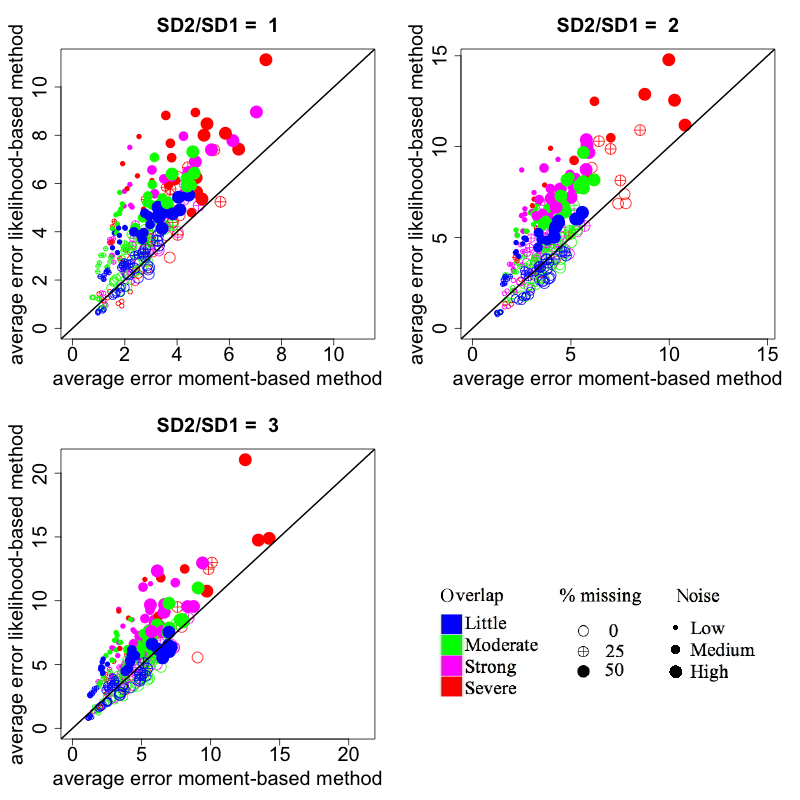 |
| --- |
| Figure S7. Comparison of the accuracy in peak summit estimation between the moment-based estimation procedure and the likelihood-based method. Each sub-plot corresponds to a different degree of asymmetry, as shown in the titles of the sub-plots (ratios between the right- and left- standard deviations). Each dot represents a simulated situation. The values were obtained by averaging the results from 100 simulations. The color represents the level of overlap between the simulated peaks. The size of the dot represents the amount of noise added to the data. The fill of the dot represents the percentage of values missing in the ion trace. |

Figure S8 shows the comparison in the estimation of the standard deviations of the peaks. The moment-based method performed better when the amount of missing was higher (solid dots).

| 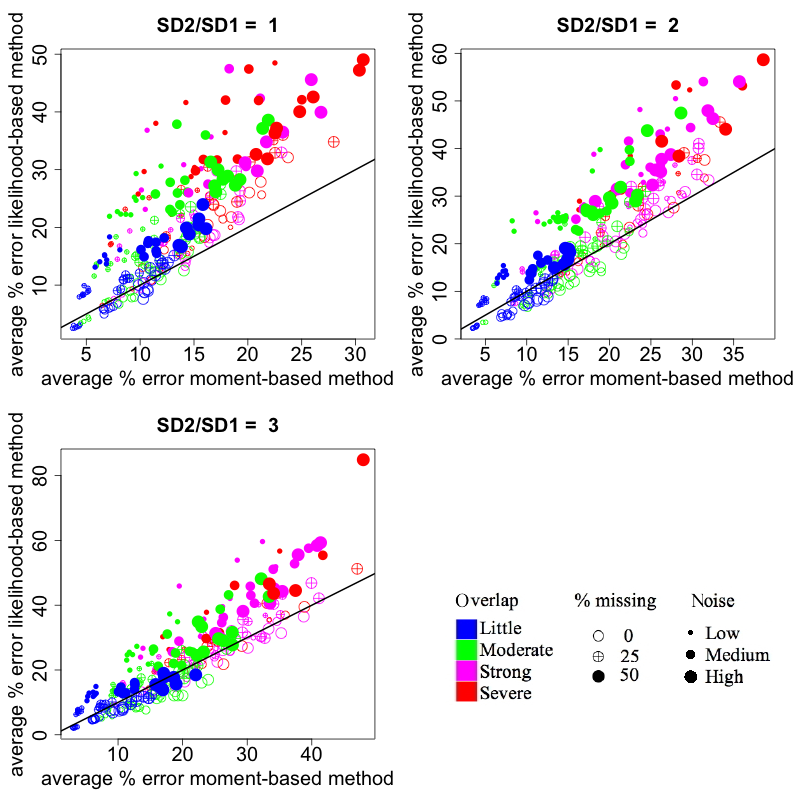 |
| --- |
| Figure S8. Comparison of the accuracy of peak standard deviation estimation between the moment-based estimation procedure and the likelihood-based method. Each sub-plot corresponds to a different degree of asymmetry, as shown in the titles of the sub-plots (ratios between the right- and left- standard deviations). Each dot represents a simulated situation. The values were obtained by averaging the results from 100 simulations. The color represents the level of overlap between the simulated peaks. The size of the dot represents the amount of noise added to the data. The fill of the dot represents the percentage of values missing in the ion trace. |
